# Assessment of the relationship between gut microbiota, inflammatory markers, and colorectal cancer

**DOI:** 10.3389/fcimb.2025.1604651

**Published:** 2025-09-09

**Authors:** Yan Cui

**Affiliations:** Gastroenterology Department, Affiliated Hospital of BeiHua University, Jilin, China

**Keywords:** colorectal cancer (CRC), gut microbiota, inflammatory biomarkers, dysbiosis, C-reactive protein, interleukin-6, tumor necrosis factor-alpha, 16S rRNA sequencing

## Abstract

**Objective:**

This study aims to evaluate the correlation between inflammation and gut microbiota characteristics in patients with colorectal cancer (CRC) through a retrospective study.

**Methods:**

This cross-sectional, non-interventional study included a total of 200 subjects, of which 150 were colorectal cancer (CRC) patients and 50 were healthy individuals. The study retrospectively reviewed hospital and laboratory archives and records from 2015 to 2020. Gut microbiota was analyzed using 16S rRNA sequencing. Inflammatory markers, including C-reactive protein (CRP), interleukin-6 (IL-6), tumor necrosis factor-alpha (TNF-α), and interleukin-beta (IL-1β), were measured using enzyme-linked immunosorbent assay (ELISA). Logistic regression analysis was used to evaluate the relationship between gut microbiota, inflammatory markers, and CRC.

**Results:**

Subjects in the colorectal cancer (CRC) group exhibited a higher proportion of Firmicutes (47.2% *vs*. 39.0%). Levels of both Firmicutes and Proteobacteria were significantly elevated in the CRC group, while Bacteroidetes levels were lower. Additionally, elevated levels of inflammatory markers were observed in the CRC group, including C-reactive protein (CRP: 9.8 mg/L *vs*. 4.1 mg/L, P<0.01), interleukin-6 (IL-6: 14.5 pg/mL *vs*. 6.2 pg/mL, P<0.01), tumor necrosis factor-alpha (TNF-α: 9.2 pg/mL *vs*. 4.3 pg/mL, P<0.01), and interleukin-1β (IL-1β: 5.8 pg/mL *vs*. 3.6 pg/mL, P<0.01). Multivariate analysis showed that higher levels of Firmicutes (OR=2.5, 95% CI: 1.4-4.5, P<0.01) and Proteobacteria (OR=2.8, 95% CI: 1.6-4.9, P<0.01) were significantly associated with an increased risk of CRC. Elevated levels of CRP (OR=3.1, 95% CI: 1.8-5.3, P<0.01) and IL-6 (OR=3.4, 95% CI: 2.0-5.8, P<0.01) were also significantly associated with an increased risk of CRC.

**Conclusion:**

There is a significant correlation between changes in gut microbiota composition and cytokine levels with the risk of colorectal cancer (CRC).

## 1 Introduction

Colorectal cancer (CRC) is one of the most common malignant tumors worldwide, with its incidence and mortality rates showing a continuous upward trend, especially in rapidly developing economies (Cardoso et al., 2021; Morgan et al., 2022; Sung et al., 2024). According to global cancer statistics, CRC is the third most common type of cancer globally and the second leading cause of cancer-related deaths (Kazemi et al., 2023). The development and progression of CRC involve multiple factors, including genetic susceptibility, environmental factors, lifestyle, and dynamic changes in the gut microbiota (Akin and Tözün, 2014; Lucas et al., 2017; Song and Chan, 2019; Silva et al., 2021). In recent years, the role of gut microbiota in the pathogenesis of CRC has become a research focus. The gut microbiota is composed of trillions of microorganisms, mainly bacteria, viruses, and fungi, whose symbiotic relationship with the host plays a crucial role in maintaining intestinal homeostasis. Dysbiosis, the imbalance in the structure and function of the microbiota, has been widely associated with the development of CRC. Research by (Huycke et al., 2002; Wang et al., 2008; Strickertsson et al., 2013; Sayed et al., 2020; Ralser et al., 2023). suggests that specific microbial communities, such as Enterococcus faecalis and Helicobacter pylori, may promote the secretion of pro-inflammatory mediators and carcinogenic metabolites, leading to DNA damage and mutations in intestinal epithelial cells, thus initiating tumorigenesis. Moreover, the dysregulation of the gut microbiota not only affects the local intestinal environment but may also promote distant tumor spread and metastasis by modulating systemic immune responses and disrupting intestinal barrier function (Ge et al., 2021; Aghamajidi and Vareki, 2022). Inflammation, as a key biological process in tumor initiation and progression, is closely linked to changes in the gut microbiota. Chronic low-grade inflammation is considered a hallmark of the tumor microenvironment, and elevated levels of inflammatory markers such as C-reactive protein (CRP), interleukin-6 (IL-6), and tumor necrosis factor-alpha (TNF-α) are often observed in CRC patients, suggesting that inflammatory responses may play a pivotal role in CRC development (Hidayat et al., 2021; Hua et al., 2021). The interaction between the microbiota and inflammation occupies a central role in CRC pathogenesis. (Yin et al., 2022). demonstrated that pathogenic bacteria in the gut can induce the release of pro-inflammatory cytokines, activating inflammatory signaling pathways such as nuclear factor kappa B (NF-κB), ultimately leading to tumor initiation and progression. Additionally, persistent inflammation results in uncontrolled proliferation and inhibited apoptosis of intestinal epithelial cells, further exacerbating malignant tumor progression. Furthermore, bacterial metabolites such as secondary bile acids and lipopolysaccharides (LPS) have been shown to directly or indirectly contribute to CRC onset and progression (Liu et al., 2022), reinforcing the complex relationship among microbiota, inflammation, and cancer. Despite current research revealing the potential links between gut microbiota and CRC, most studies have focused on individual microbial species, with small sample sizes and a lack of comprehensive, systematic evaluations of microbiota structure and function. Moreover, the specific mechanisms of interaction between inflammatory markers and the gut microbiota in CRC, particularly how they influence CRC through the regulation of host immune responses, remain unclear. Thus, further investigation into the relationship between gut microbiota and inflammatory markers in CRC is of significant scientific importance in understanding the mechanisms underlying CRC. This study aims to comprehensively analyze the gut microbiota using high-throughput sequencing technology and, in conjunction with the measurement of inflammatory marker levels, evaluate the synergistic effects of both in CRC, further exploring the specific mechanisms of the microbiota-inflammation axis in tumorigenesis.

## 2 Research methods

### 2.1 Study design

This study is a retrospective, cross-sectional, non-interventional study aimed at evaluating the relationship between gut microbiota and inflammatory markers, as well as their correlation with colorectal cancer (CRC). The study utilized data from CRC patients and healthy control groups collected between 2015 and 2020. Through multi-stage data collection and analysis, the study ensured comprehensiveness and reliability.

### 2.2 Study setting

The data for this study were sourced from a large teaching hospital. All study subjects were patients treated at this hospital, and the data were obtained through the hospital’s electronic medical records (EMR) system and laboratory databases. The study period spanned from January 2015 to December 2020, covering both outpatient and inpatient populations.

### 2.3 Study subjects

**Figure 1:**
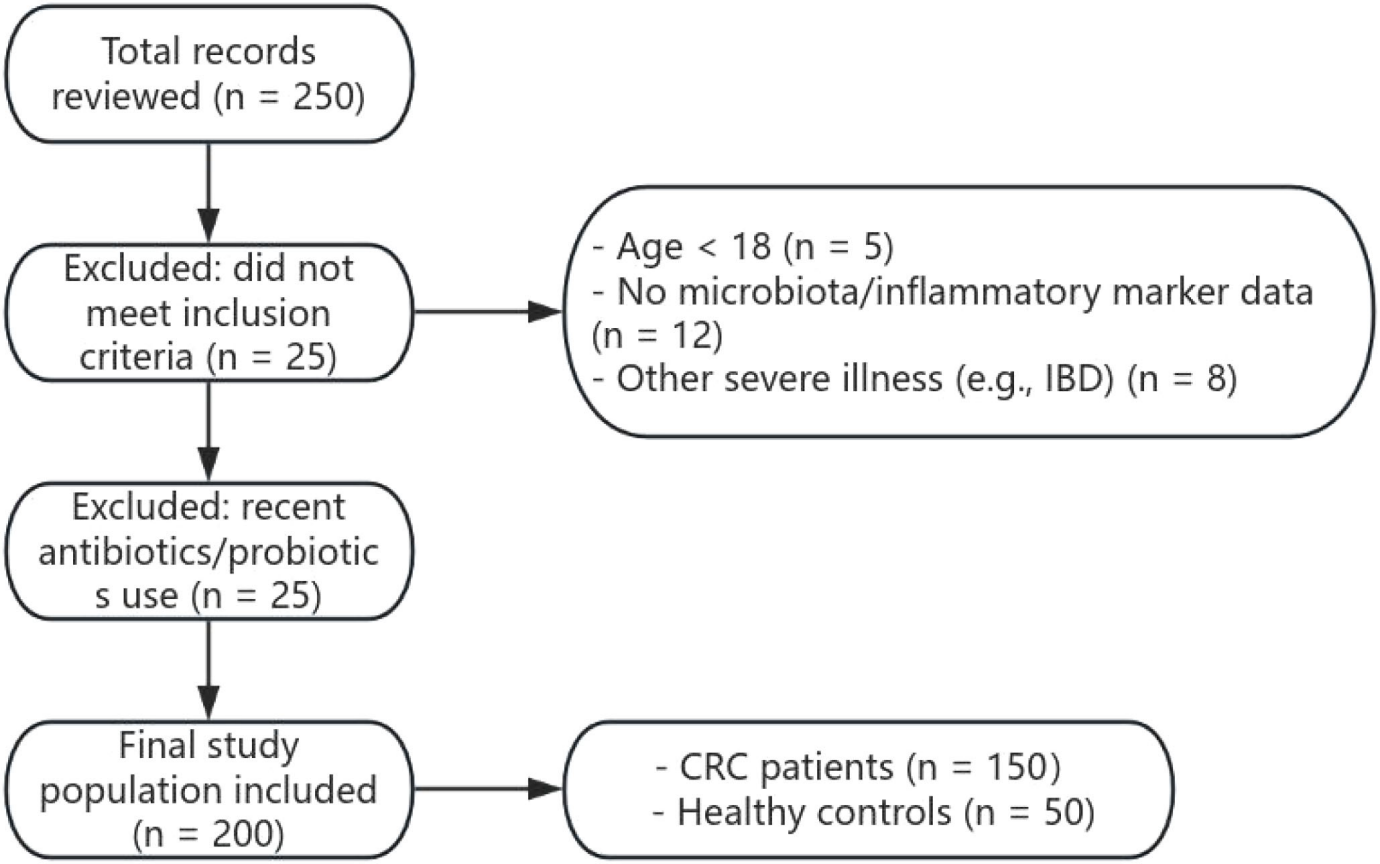
Flow diagram of subject selection and exclusion.

A total of 200 subjects were included in this study, comprising 150 patients diagnosed with colorectal cancer (CRC) and 50 healthy controls. Inclusion criteria were: (1) age ≥ 18 years; (2) diagnosed with colorectal cancer, with complete clinical and laboratory records; (3) availability of gut microbiota and inflammatory marker test data. Exclusion criteria were: (1) received antibiotics, probiotics, or other treatments affecting gut microbiota within 5 months prior to sampling; (2) missing key clinical information; (3) presence of other severe conditions affecting gut microbiota or inflammatory markers, such as inflammatory bowel disease (IBD). The subject enrollment process is shown in **Figure 1**.

### 2.4 Data collection and analysis

#### 2.4.1 Data collection

Gut microbiota data were obtained through 16S rRNA gene sequencing, with stool samples collected from patients. The sequencing process included DNA extraction from the samples, amplification, and high-throughput sequencing. Sequencing data were analyzed using the QIIME2 software to identify taxonomic classifications such as phylum, class, order, family, and genus.

Serum concentrations of inflammatory markers including C-reactive protein (CRP), interleukin-6 (IL-6), tumor necrosis factor-α (TNF-α), and interleukin-1β (IL-1β) were measured using enzyme-linked immunosorbent assay (ELISA) kits provided by Roche Diagnostics (Basel, Switzerland), in accordance with the manufacturer’s protocols. All samples were analyzed in duplicate, and the average values were used for statistical analysis. Inter- and intra-assay coefficients of variation were <10% for all kits.

#### 2.4.2 Statistical analysis

Statistical analysis was performed using SPSS version 25.0, with a P-value < 0.05 considered statistically significant.

The specific analysis steps were as follows:

(1) Descriptive Statistics: Demographic and baseline characteristics of the subjects were analyzed using descriptive statistics such as mean and standard deviation.

Group Comparison: Differences in gut microbiota composition and inflammatory markers between groups were compared using the t-test or Mann-Whitney U test.

(2)Correlation Analysis: Since most variables were not normally distributed, Spearman correlation coefficients (two-tailed) were used to evaluate the correlations between gut microbiota abundance and inflammatory marker levels.

(3) Multivariate Regression Analysis: Logistic regression was used to evaluate the association between microbiota characteristics, inflammatory markers, and CRC risk, adjusting for potential confounding factors such as age, gender, and BMI.

(4) Participants were stratified into “high” and “low” groups for each inflammatory marker and selected microbiota taxa based on the median value across the entire cohort (CRC + Control), unless otherwise specified. This median-based dichotomization allowed for non-parametric comparisons and subgroup analysis.

(5)Sensitivity Analysis: To assess the robustness of the primary findings, sensitivity analyses were conducted using multivariate logistic regression under four different scenarios: excluding current smokers, including only early-stage CRC patients (Stage I–II), adjusting for body mass index (BMI), and adjusting for family history of CRC. For each scenario, odds ratios (ORs) and 95% confidence intervals (CIs) were recalculated. All tests were two-tailed, and a P value < 0.05 was considered statistically significant.

#### 2.4.3 Microbiota DNA extraction and 16S rRNA gene sequencing

Fecal samples were collected and stored at –80°C until further processing. Bacterial genomic DNA was extracted using the QIAamp DNA Stool Mini Kit (QIAGEN, Hilden, Germany), following the manufacturer’s instructions. DNA quantity and purity were assessed using a NanoDrop 2000 spectrophotometer (Thermo Fisher Scientific, USA).The V3–V4 hypervariable regions of the bacterial 16S rRNA gene were amplified using universal primers 341F (5′-CCTACGGGNGGCWGCAG-3′) and 806R (5′-GGACTACHVGGGTATCTAAT-3′) with sample-specific barcodes. PCR amplification was carried out in triplicate with Phusion High-Fidelity DNA Polymerase (New England Biolabs, USA). PCR products were purified using AMPure XP beads (Beckman Coulter, USA) and quantified using a Qubit 3.0 Fluorometer (Invitrogen, USA).Amplicon libraries were constructed using the TruSeq DNA PCR-Free Library Preparation Kit (Illumina, San Diego, USA) and sequenced on the Illumina MiSeq platform (2 × 300 bp paired-end reads). Raw reads were processed using QIIME2 (version 2022.2), including demultiplexing, quality filtering, and removal of chimeric sequences. Amplicon sequence variants (ASVs) were inferred using the DADA2 algorithm and taxonomic assignment was performed against the SILVA 138 database.

### 2.5 Bias control

To minimize selection bias and information bias, the study implemented the following measures:

1. Selection Bias: Strict inclusion and exclusion criteria were applied to ensure homogeneity among study subjects and to avoid confounding factors.
2. Information Bias: All data were extracted and reviewed by well-trained researchers to ensure consistency and accuracy. The data extraction process followed standardized procedures and was reviewed at multiple time points to ensure reliability.
3. Adjustment for Confounding Factors: In the multivariate regression analysis, potential confounding factors such as age, gender, BMI, and smoking status were considered to mitigate their impact on the study results.

### 2.6 Sample size

The sample size was determined based on power analysis from previous studies (Zackular et al., 2014; Wong et al., 2017). Assuming a significant difference in gut microbiota and inflammatory markers between the CRC group and the control group (effect size of 0.5), a total of 200 subjects were included to achieve 80% statistical power and a 5% significance level (alpha). Of these, 150 were CRC patients and 50 were healthy controls.

### 2.7 Ethical approval

This study was reviewed and approved by the Ethics Committee of the Affiliated Hospital of BeiHua University (Approval No. 2024-CRC-051). Due to the retrospective design and the use of anonymized clinical and laboratory data, the requirement for written informed consent was waived by the Ethics Committee. All study procedures were carried out in accordance with the ethical standards of the Declaration of Helsinki and relevant institutional guidelines.

## 3 Results

### 3.1 Comparison of baseline characteristics

The average age of the CRC group was 63.1 years, while that of the control group was 60.0 years. In terms of smoking status, the proportion of current smokers was significantly higher in the CRC group (23.3% *vs*. 10%, P=0.03). However, there were no statistically significant differences in gender distribution and BMI between the two groups. Smoking status did not significantly affect gut microbial composition or inflammatory marker levels in either group (all P > 0.05), and thus was not considered a confounding factor in this study.

Additional subgroup analyses indicated that age, sex, and BMI did not significantly affect gut microbiota profiles or inflammatory marker levels (P > 0.05), suggesting that the core findings are robust across demographic strata. See **Table 1**.

**Table 1:** omparison of baseline characteristics.

Characteristic	All participants (n=200)	CRC group (n=150)	Control group (n=50)	P-value
Age (years)	62.3 ± 10.4	63.1 ± 9.8	60.0 ± 11.7	0.12
Gender				
Male	110 (55%)	85 (56.7%)	25 (50%)	0.48
Female	90 (45%)	65 (43.3%)	25 (50%)	0.48
BMI (kg/m2)	26.7 ± 4.3	27.0 ± 4.2	25.8 ± 4.5	0.14
Smoking status				
Current smokers	40 (20%)	35 (23.3%)	5 (10%)	0.03
Former smokers	60 (30%)	45 (30%)	15 (30%)	1.00
Never smoked	100 (50%)	70 (46.7%)	30 (60%)	0.09
Family history of CRC	50 (25%)	40 (26.7%)	10 (20%)	0.40
CRC stage				
Stage I	30 (20%)	30 (20%)	N/A	N/A
Stage II	45 (30%)	45 (30%)	N/A	N/A
Stage III	45 (30%)	45 (30%)	N/A	N/A
Stage IV	30 (20%)	30 (20%)	N/A	N/A

### 3.2 Descriptive data on gut microbiota characteristics

**Figure 2:**
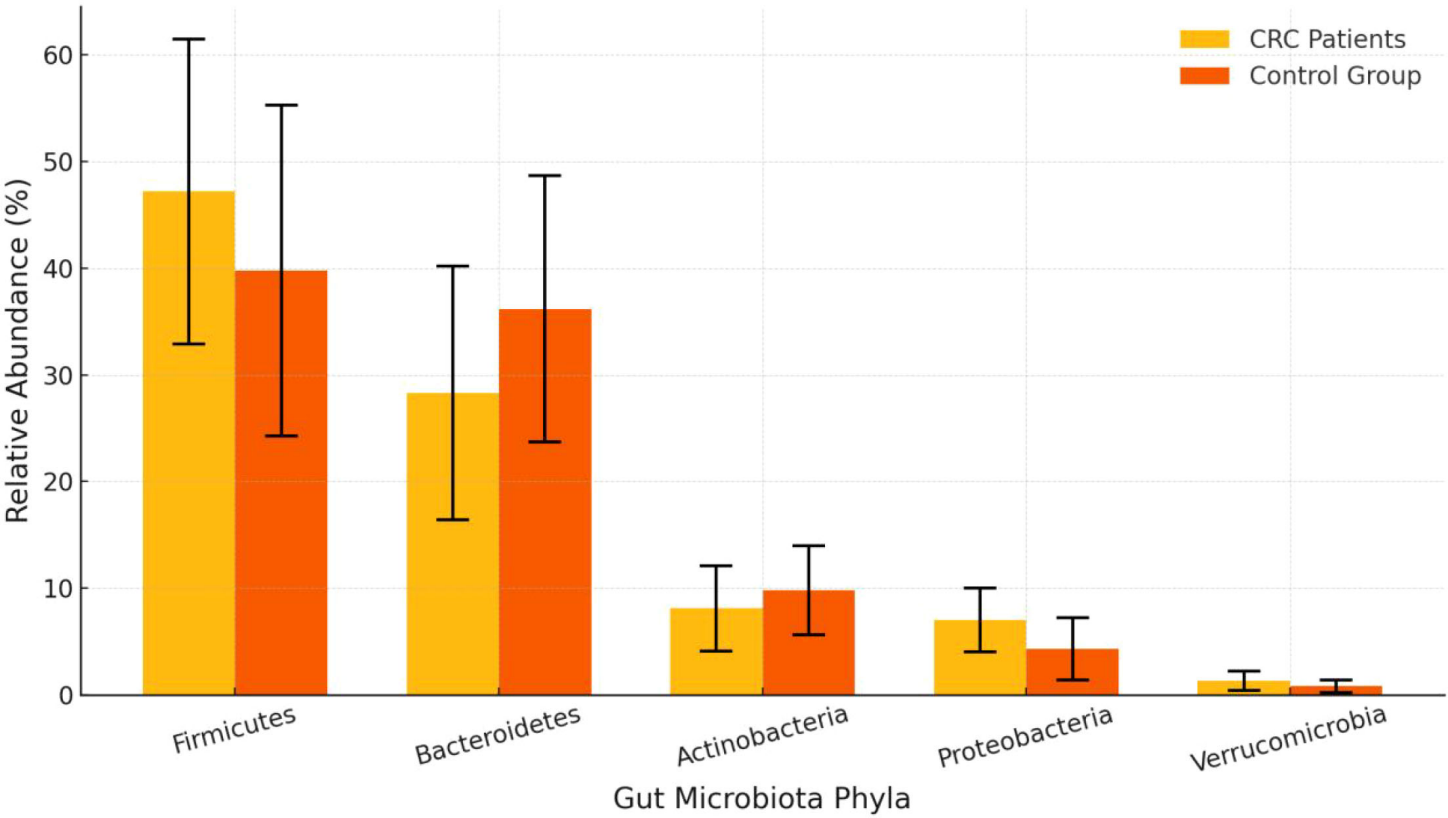
Gut microbiota composition bar chart.

**Figure 3:**
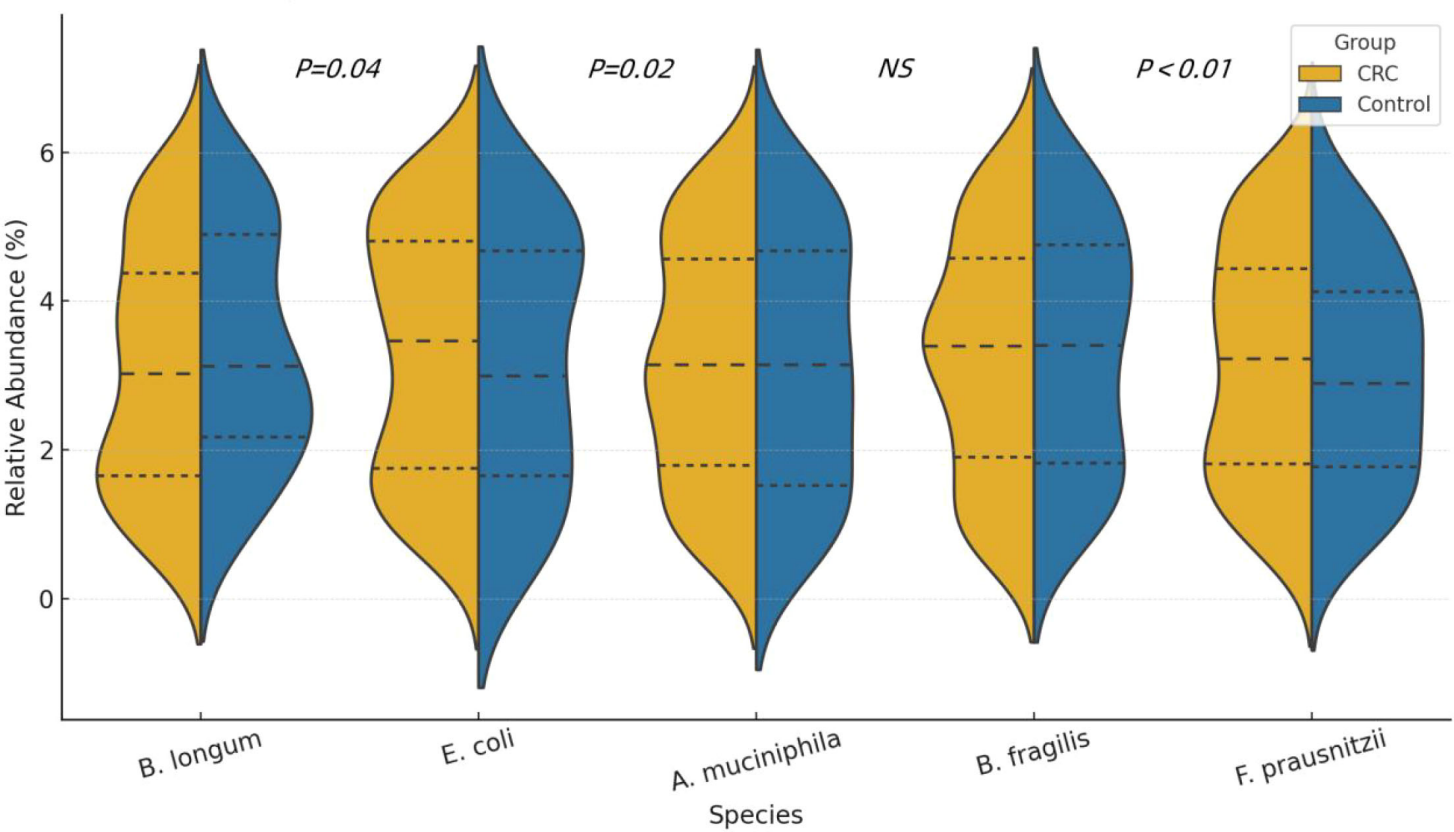
Violin plots showing the distribution of relative abundance (%) for five representative bacterial species in CRC patients and healthy controls. The plots display the median and interquartile ranges (IQR) for each group.

The proportion of Firmicutes in the CRC group was significantly higher than in the control group (47.2% *vs*. 39.8%, P = 0.01), while the proportion of Bacteroidetes was significantly lower (28.3% *vs*. 36.2%, P < 0.01). Additionally, the proportions of Proteobacteria and Verrucomicrobia were also notably elevated in the CRC group, with statistically significant differences. See **Table 2** and **Figures 2**, **3**.

**Table 2:** Descriptive data on gut microbiota characteristics.

Gut microbiota phyla	All participants (n=200)	CRC group (n=150)	Control group (n=50)	P-value
Firmicutes (%)	45.3 ± 15.1	47.2 ± 14.3	39.8 ± 15.5	0.01
Bacteroidetes (%)	30.5 ± 12.4	28.3 ± 11.9	36.2 ± 12.5	<0.01
Actinobacteria (%)	8.5 ± 4.1	8.1 ± 4.0	9.8 ± 4.2	0.04
Proteobacteria (%)	6.3 ± 3.2	7.0 ± 3.0	4.3 ± 2.9	<0.01
Verrucomicrobia (%)	1.2 ± 0.8	1.3 ± 0.9	0.8 ± 0.6	<0.01
Others (%)	8.2 ± 5.1	8.1 ± 4.9	9.1 ± 5.5	0.27

### 3.3 Descriptive data on inflammatory markers

**Figure 4:**
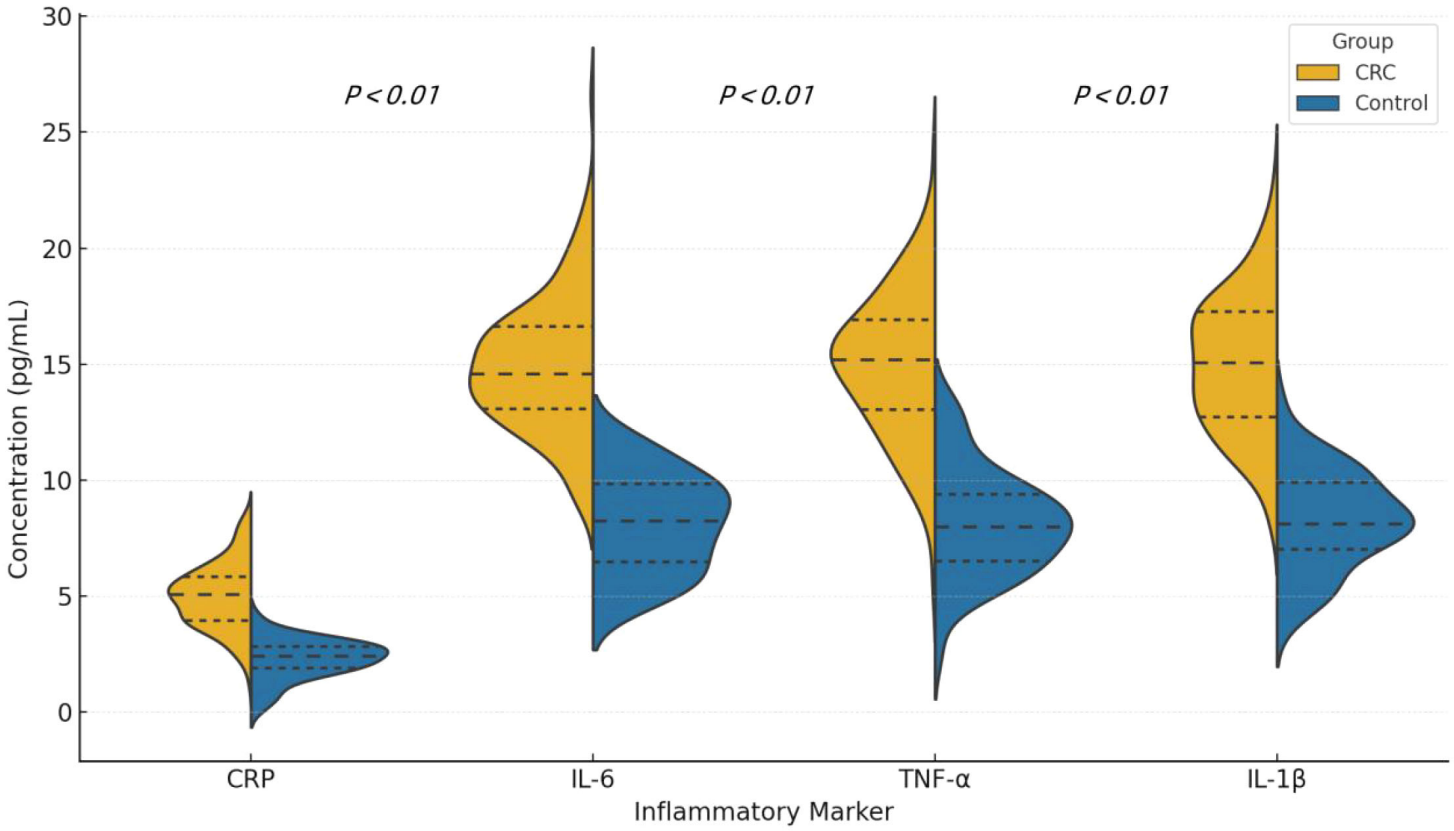
Violin plots showing the distribution of serum levels of CRP, IL-6, TNF-α, and IL-1β in colorectal cancer (CRC) patients and healthy controls. Each plot displays the median and interquartile range (IQR). P values were calculated using the Mann-Whitney U test.

The levels of CRP, IL-6, TNF-α, and IL-1β were significantly higher in the CRC patient group compared to the control group (CRP: 9.8 mg/L *vs*. 4.1 mg/L, P < 0.01; IL-6: 14.5 pg/mL *vs*. 6.2 pg/mL, P < 0.01). These results indicate a higher level of systemic inflammation in the CRC patient group. See **Table 3** and **Figure 4**.

**Table 3:** Descriptive data on inflammatory markers.

Inflammatory marker	All participants (n=200)	CRC group (n=150)	Control group (n=50)	P-value
CRP (mg/L)	8.4 ± 5.2	9.8 ± 5.3	4.1 ± 2.8	<0.01
IL-6 (pg/mL)	12.3 ± 8.7	14.5 ± 9.1	6.2 ± 4.3	<0.01
TNF-α (pg/mL)	7.9 ± 4.8	9.2 ± 5.0	4.3 ± 2.7	<0.01
IL-1β (pg/mL)	5.2 ± 3.6	5.8 ± 3.7	3.6 ± 2.8	<0.01

### 3.4 Association between gut microbiota and inflammatory markers

The analysis revealed that an increase in Firmicutes was significantly associated with elevated CRP levels (OR=1.04, 95% CI: 1.01-1.07, P=0.02). A reduction in Bacteroidetes was associated with lower levels of inflammatory markers (OR=0.95, 95% CI: 0.92-0.98, P < 0.01). Additionally, an increase in the proportion of Proteobacteria showed a positive correlation with elevated levels of both IL-6 and CRP (See **Table 4**).

**Table 4:** Association between gut microbiota and inflammatory markers.

Variable	OR	95%CI	P-value
Firmicutes (%)	1.04	1.01-1.07	0.02
Bacteroidetes (%)	0.95	0.92-0.98	<0.01
Actinobacteria (%)	1.07	0.99-1.15	0.08
Proteobacteria (%)	1.10	1.03-1.17	<0.01
Verrucomicrobia (%)	1.25	1.07-1.47	<0.01
CRP (pg/mL)	1.12	1.05-1.20	<0.01
IL-6(pg/mL)	1.15	1.09-1.21	<0.01
TNF-α(pg/mL)	1.18	1.09-1.27	<0.01
IL-1β(pg/mL)	1.20	1.10-1.31	<0.01

**Figure 5:**
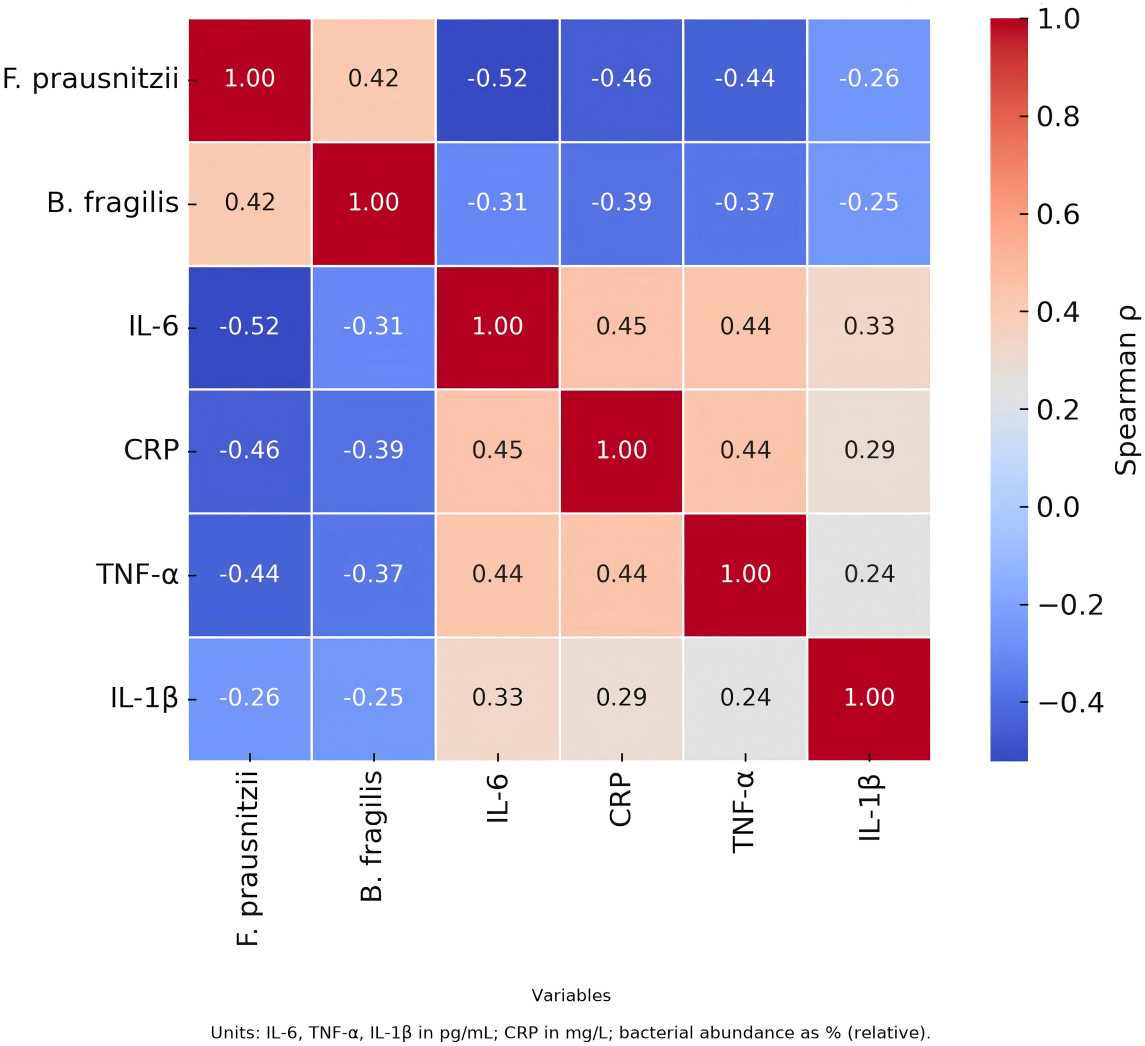
Correlation between gut microbiota and inflammatory markers.

Correlation analysis demonstrated an inverse relationship between F. prausnitzii and both IL-6 and CRP, while B. fragilis was positively correlated with IL-6, suggesting potential pro- and anti-inflammatory roles of these taxa, respectively. See **Figure 5**.

### 3.5 Odds ratios for colorectal cancer

**Figure 6:**
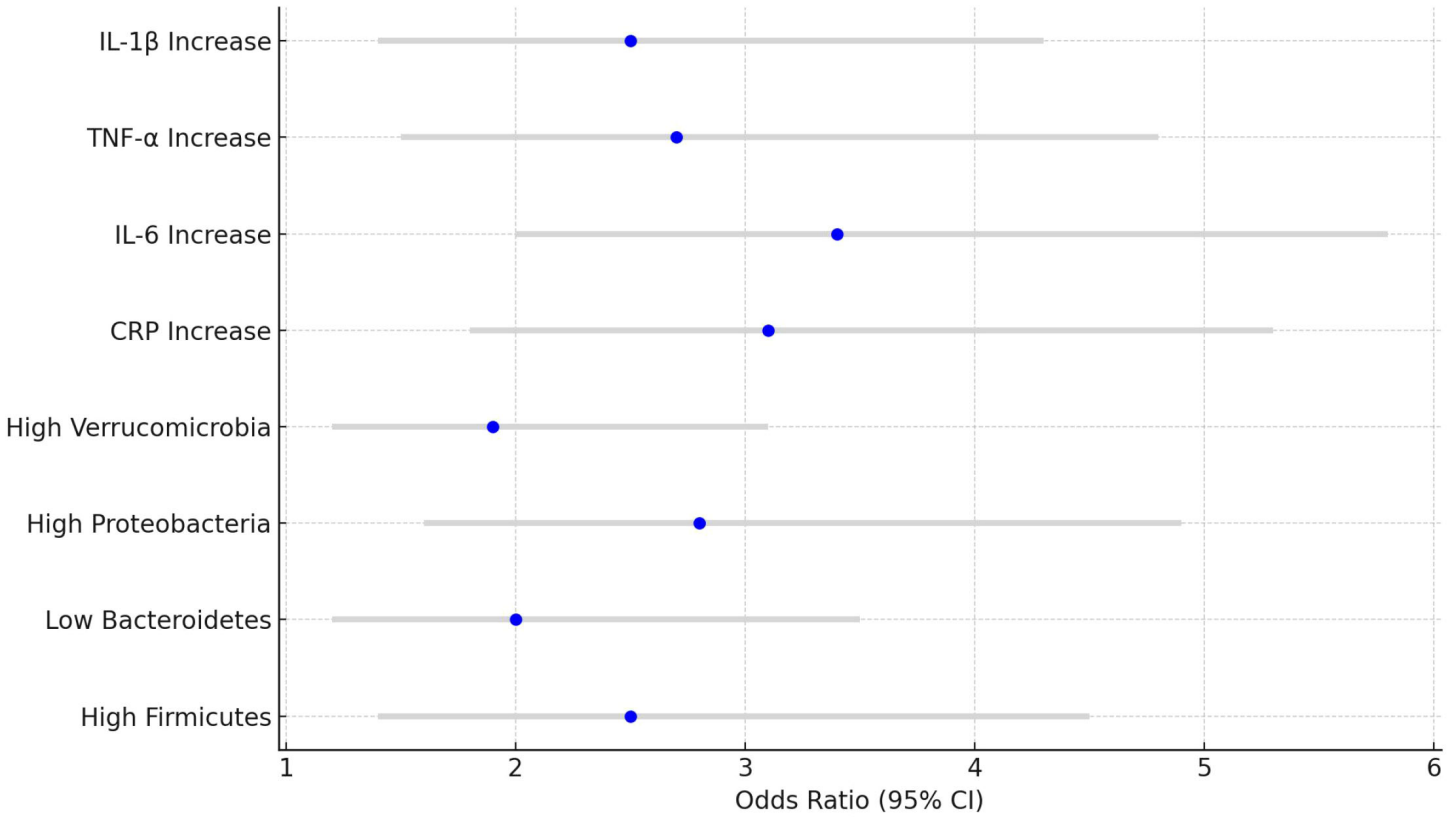
Colorectal cancer risk forest plot.

Higher levels of Firmicutes and Proteobacteria were significantly associated with an increased risk of CRC (Firmicutes OR=2.5, 95% CI: 1.4-4.5, P<0.01; Proteobacteria OR=2.8, 95% CI: 1.6-4.9, P<0.01). Additionally, elevated levels of CRP and IL-6 were also significantly associated with a higher risk of developing CRC (CRP OR=3.1, 95% CI: 1.8-5.3, P<0.01; IL-6 OR=3.4, 95% CI: 2.0-5.8, P<0.01). See **Table 5** and **Figure 6**.

**Table 5:** Odds ratios (OR) for colorectal cancer (CRC).

Variable	OR	95%CI	P-value
High Firmicutes (%)	2.5	1.4-4.5	<0.01
Low Bacteroidetes (%)	2.0	1.2-3.5	<0.01
High Proteobacteria (%)	2.8	1.6-4.9	<0.01
High Verrucomicrobia (%)	1.9	1.2-3.1	<0.01
Elevated CRP (pg/mL)	3.1	1.8-5.3	<0.01
Elevated IL-6 (pg/mL)	3.4	2.0-5.8	<0.01
Elevated TNF-α (pg/mL)	2.7	1.5-4.8	<0.01
Elevated IL-1β (pg/mL)	2.5	1.4-4.3	<0.01

### 3.6 Sensitivity analysis

**Table 6** presents the results of the sensitivity analysis, conducted to assess the robustness of the primary conclusions. In this analysis, smoking status was excluded, and the study was limited to early-stage colorectal cancer (CRC) patients. Adjustments were made for factors such as body mass index (BMI) and family history of CRC. The adjusted odds ratios (OR) and 95% confidence intervals (CI) are shown below. The results indicate that the main significant associations remained robust across different adjustment scenarios.

**Table 6:** Sensitivity analysis.

Sensitivity analysis	OR	95%CI	P-value
Excluding smokers	2.3	1.3-4.0	<0.01
Including only early-stage CRC patients	2.1	1.2-3.7	<0.01
Adjusting for BMI	2.4	1.4-4.2	<0.01
Adjusting for family history of CRC	2.5	1.4-4.3	<0.01

Early-stage CRC was defined as Stage I and Stage II based on the AJCC 8th edition staging system.

## 4 Discussion

CRC is one of the most common malignancies worldwide, with a complex pathophysiological mechanism, insidious progression, and rapid advancement. The clinical presentation of colorectal cancer is diverse, with early symptoms often being nonspecific. Patients may experience intermittent abdominal pain, changes in bowel habits, abnormal stool shape, or blood in the stool (Holtedahl et al., 2021; Moullet et al., 2022; Fritz et al., 2023). As the disease progresses, patients may develop abdominal distension, bowel obstruction, weight loss, anemia, and other systemic symptoms, while advanced-stage patients may experience distant metastasis of cancer cells. The pathogenesis of colorectal cancer is not yet fully understood, but extensive research suggests that gut dysbiosis, chronic inflammation, and genetic mutations play crucial roles. The gut microbiota is key in maintaining host immune homeostasis, metabolic balance, and epithelial barrier integrity. When the gut microbiota structure is altered, harmful bacteria such as Clostridium and Enterococcus faecalis can overgrow, while beneficial bacteria like Bifidobacteria and Lactobacilli decrease, leading to disruption of gut barrier function. This increases the secretion of inflammatory factors such as IL-6 and TNF-α, creating a chronic low-grade inflammatory microenvironment that further activates signaling pathways such as nuclear factor-κB (NF-κB) and Janus kinase/signal transducer and activator of transcription (JAK/STAT). This, in turn, induces the proliferation and invasion of tumor cells, ultimately promoting the development and progression of colorectal cancer (Zhu et al., 2020; Chattopadhyay et al., 2021; Wang et al., 2021). If timely diagnosis and treatment are not provided, the prognosis for patients is usually poor, and the cancer may rapidly advance to intermediate or late stages, with distant metastases (e.g., liver or lung metastasis), ultimately leading to a significant increase in cancer-related mortality.

Numerous studies have investigated the relationship between gut microbiota and colorectal cancer (CRC), consistently reporting alterations in microbial diversity and composition in CRC patients. For example, previous work has highlighted increased abundance of Fusobacterium nucleatum and decreased Bifidobacterium in CRC cohorts (Miyasaka et al., 2024). Our study supports these observations by confirming significant differences in the relative abundance of major phyla, including Firmicutes, Bacteroidetes, and Proteobacteria. However, a distinguishing feature of our study is the integration of microbiota profiling with systemic inflammatory markers (CRP, IL-6, TNF-α, and IL-1β) in the same CRC population. This dual assessment enabled us to explore the potential mechanistic link between microbial dysbiosis and inflammation, a critical pathway in tumor initiation and progression. Furthermore, our analysis stratified microbiota profiles by clinical variables such as disease stage and family history, offering a more nuanced understanding of host-microbe interactions in CRC pathogenesis. While numerous studies have independently addressed the roles of the gut microbiome or inflammatory factors in CRC, our study offers a more integrated perspective by evaluating both dimensions within the same clinical cohort (Chen et al., 2021; Gao et al., 2022). This simultaneous profiling enables a more direct assessment of the potential interplay between microbial dysbiosis and systemic inflammation in CRC pathogenesis. Additionally, we conducted subgroup analyses stratified by tumor stage and family history of CRC, revealing distinct microbial patterns and inflammatory responses in these subsets. These findings help refine our understanding of individual variability in CRC development. Importantly, we also report correlation patterns between dominant microbial phyla and specific inflammatory markers (e.g., positive correlation between Proteobacteria and IL-6), which provides novel insights into the microbiota–inflammation axis. This integrated approach advances the current understanding beyond descriptive studies and highlights the potential for combined microbial and immunological biomarkers in CRC.

This study systematically explored the complex relationship between gut microbiota composition and colorectal cancer (CRC) pathogenesis by analyzing the gut microbiota and inflammatory markers of CRC patients and healthy controls. It highlighted the critical roles of dysbiosis and systemic inflammation in the pathophysiology of CRC. The findings showed a significant increase in the relative abundance of Firmicutes and Proteobacteria, along with a decrease in Bacteroidetes in the gut microbiota of CRC patients, consistent with previous research. The overgrowth of Firmicutes and Proteobacteria is closely linked to intestinal inflammation, impaired barrier function, and cancer development, while the reduction in Bacteroidetes may diminish the anti-inflammatory capacity of the gut, making the tumor microenvironment more susceptible to inflammatory mediators, further promoting cancer progression. The increase in Firmicutes was associated with metabolic dysfunction and reduced production of short-chain fatty acids (SCFAs), which enhances pro-inflammatory responses. These bacteria can activate the Toll-like receptor (TLR) signaling pathway through their metabolic products, such as lipopolysaccharides (LPS), triggering the activation of nuclear factor κB (NF-κB) and leading to increased secretion of pro-inflammatory cytokines, including TNF-α, IL-6, and CRP, which are strongly associated with a higher risk of CRC (Sheikh et al., 2021; Panyathep et al., 2023). Elevated levels of IL-6, TNF-α, and CRP in CRC patients suggest that systemic inflammation is a core driver of CRC development. IL-6 promotes tumor cell proliferation and inhibits apoptosis by activating the Janus kinase (JAK)-signal transducer and activator of transcription 3 (STAT3) pathway, further exacerbating CRC progression. TNF-α enhances tumor cell invasiveness through the NF-κB pathway and promotes epithelial-mesenchymal transition (EMT), granting cancer cells migratory and metastatic potential, which are critical in CRC progression and metastasis (Bakshi et al., 2022). CRP, as an acute-phase protein, not only marks increased inflammation in CRC patients but also exacerbates local and systemic inflammation by modulating the function of vascular endothelial cells and immune cells. The development of CRC involves not only microbial dysbiosis and inflammation but also complex gene-environment interactions and cross-regulation among multiple cellular signaling networks. This study, through retrospective cohort analysis, was the first to establish a clear positive association between the increase in Firmicutes and Proteobacteria, the decrease in Bacteroidetes, and the elevated levels of inflammatory markers such as IL-6, TNF-α, and CRP with the incidence of CRC. Although the study revealed a strong association between gut dysbiosis and CRC, the causal relationship remains unclear, and future prospective longitudinal studies are needed to validate these findings and investigate how microbial metabolites regulate the host immune microenvironment and influence CRC progression. Moreover, the study pointed out that modulating the gut microbiota, particularly through interventions with probiotics, prebiotics, or dietary fiber, could be a promising strategy for primary prevention of CRC. Probiotics may help restore gut microbial balance by promoting SCFA production, reducing pro-inflammatory responses, and lowering the risk of tumor development by preserving intestinal barrier function (Mahdavi et al., 2021; Fusco et al., 2023). The findings of this study provide new insights for the early diagnosis, prognosis assessment, and personalized treatment of CRC.

Recent studies have highlighted the significant role of genetic factors in the pathogenesis of CRC, in addition to environmental and microbial influences (Liu et al., 2020; Yang et al., 2020). Common susceptibility genes include APC, KRAS, TP53, and mismatch repair (MMR) genes such as MLH1 and MSH2. Mutations in these genes contribute to tumor initiation and progression through diverse mechanisms. For instance, APC mutations often lead to aberrant activation of the Wnt/β-catenin pathway, whereas defects in MMR genes are strongly associated with Lynch syndrome (Parker and Neufeld, 2020; Wang et al., 2024). Moreover, some evidence suggests that genetic mutations may alter the gut microbiota composition or modulate local inflammation, potentially linking host genetics to microbial dysbiosis and immune response (Lee et al., 2022; Gates et al., 2024). These interactions warrant further investigation to clarify their contributions to CRC development.

Despite the important findings of this study, there are several limitations. First, the study employed a retrospective cross-sectional design, making it difficult to establish causality. Future prospective studies are needed to validate these findings. Second, the sample size was relatively small, which may limit the generalizability of the results. Future research should involve larger population cohorts and consider other variables that may influence gut microbiota and inflammation, such as diet and lifestyle factors. Additionally, this study relied on 16S rRNA sequencing to analyze gut microbiota composition. While this method provides an overview of microbial communities, it does not allow for an in-depth analysis of functional genomics. Future studies should incorporate metagenomic sequencing to further uncover how these microbes influence CRC development and progression through metabolic pathways.

This study revealed the important role of gut microbiota dysbiosis and systemic inflammation in the development of colorectal cancer (CRC). The increase in Firmicutes and Proteobacteria, the decrease in Bacteroidetes, and the elevation of inflammatory markers such as CRP and IL-6 were all significantly associated with an increased risk of CRC. These findings offer new insights into the potential of preventing and managing CRC by modulating the gut microbiota and controlling inflammatory responses. Future longitudinal and interventional studies will further clarify the clinical application of these biomarkers and provide a foundation for the development of personalized cancer treatment strategies.

## Data Availability

The raw data supporting the conclusions of this article are available in the Zenodo repository at: https://doi.org/10.5281/zenodo.16900478.
